# Effects of Serum Lipids on the Long-Term Prognosis of Ampullary Adenocarcinoma Patients after Curative Pancreatoduodenectomy

**DOI:** 10.3390/curroncol29110706

**Published:** 2022-11-21

**Authors:** Zheng Li, Xiaojie Zhang, Chongyuan Sun, He Fei, Zefeng Li, Dongbing Zhao

**Affiliations:** Department of Pancreatic and Gastric Surgical Oncology, National Cancer Center/National Clinical Research for Cancer/Cancer Hospital, Chinese Academy of Medical Sciences and Peking Union Medical College, Beijing 100021, China

**Keywords:** ampullary adenocarcinoma, serum lipids, lipid metabolism, prognosis, survival

## Abstract

Background: Serum lipids (SLs), the prominent indicators of lipid metabolism, produce an intricate impact on proliferation, invasion, and metastasis of cancer cells. However, the effects of serum lipids on the prognosis of ampullary adenocarcinoma (AC) have not been investigated. Methods: Patients with AC in the National Cancer Center of China between January 1998 and December 2020 were retrospectively reviewed. Survival analysis for overall survival (OS, Time from operation to death) and recurrence-free survival (RFS, Time from operation to first-time recurrence) was performed using Kaplan–Meier analysis and Cox proportional hazards models. Results: A total of 232 AC patients were enrolled into the study. SLs levels were significantly lower in patients with vascular invasion compared to those without (all *p* < 0.05). The 1-year, 3-year, and 5-year OS rates for AC patients were 86.1%, 64.1%, and 47.6% and 75.8%, 54.8%, and 46.5% for RFS. Biliary/pancreatic fistula (31.9%) and chemotherapy (81.4%) were the majority of postoperative complications and adjuvant therapy, respectively. According to Cox analysis, preoperative LDL-C was an independent prognostic factor for RFS (HR = 0.43, 95% CI: 0.21–0.85, *p* = 0.015), whereas no statistical significance existed in the analysis of HDL-C, TC, and TG. Conclusions: High levels of preoperative LDL-C is a significant predictor of prolonged prognosis in AC patients, which was also observed to be a protective factor to reduce vascular invasion.

## 1. Introduction

Ampullary adenocarcinoma (AC) is a rare malignancy with an incidence of 0.73 cases/100,000 inhabitants, and only accounts for 0.2% of gastrointestinal tumors [1,2]. It is suggested that AC is more frequent in male patients, with a wide age range of diagnoses [2]. So far, surgical treatment, including the whipple procedure, is still the main curative strategy for AC patients. The 5-year survival rate of AC is lower than 45% in the resected patients, which indicates a heavy disease burden on patients with AC [3,4].

In recent years, lipid metabolism in cancer has attracted a lot of attention [5]. Lipids are hydrophobic molecules and consist of sterols, monoglycerides, diacylglycer-ides, triglycerides, phospholipids, and glycolipids. A number of lipids are originated from fatty acids (FAs), a diverse group of molecules including long hydrocarbon chains varying in length (number of carbon atoms) and saturation (number of double bonds) [6]. As one of the most prominent metabolic alterations in cancer, dysregulation in lipid metabolism produces a vital impact on the energy acquisition, biological membrane formation, proliferation, survival, invasion, metastasis, and drug resistance of cancer cells [7,8,9]. In daily clinical work, Serum lipids function as indicators to evaluate the status of patient lipid metabolism, including triglyceride (TG), total cholesterol (TC), low-density lipoprotein cholesterol (LDL-C), and high-density lipoprotein cholesterol (HDL-C). Currently, several studies have determined that serum lipids aforementioned are significantly associated with the tumor progression and therapy in multiple cancer types, including lung cancer [10], liver cancer [11], breast cancer [12], prostate cancer [13], gastric cancer [14] colorectal cancer [15,16], and ovarian cancer [17]. However, the exact impact of serum lipids on prognosis of AC patients is still unclear.

Given these considerations, the discussion on the influence of lipid metabolism on the AC patient survival can specify potential strategies for lipid metabolism, thus providing new insights for AC arrangement. As such, we conducted this study to determine the association between serum lipids and long-term prognosis (OS and RFS) of AC.

## 2. Materials and Methods

### 2.1. Patients and Study Design

The present study was a retrospective one of pathologically confirmed AC patients in the China National Cancer Center between January 1998 and December 2020. All these patients were operated on for curative purposes for malignant ampullary adenocarcinoma. Computer Tomography (CT) was the main way to detect tumors preoperatively. At the same time, endoscopy was also a powerful means of detecting tumors before surgery. The surgical informed consent forms of these patients were signed generally. Moreover, the study was approved by the institutional review board of the China National Cancer Center. The levels of serum lipids (TC, TG, HDL-C, and LDL-C) were detected by the laboratory of the center preoperatively. In addition, the clinicopathologic characteristic data of AC patients were reviewed and collected accurately from the medical record system. The exclusion criteria were as follows: (1) patients with a history of other tumors; (2) patients with positive surgical margins; (3) patients with missed lymph node resection data; (4) patients whose clinicopathologic information was missed; (5) patients whose information of postoperative adjuvant treatment were missed; (6) patients whose survival information was unknown; and (7) patients who had used lipid-lowering drugs. According to the optimal cutoff values for the serum lipids determined by ROC analysis, patients were divided into a high serum lipids group and a low serum lipids group. Finally, a total of 232 patients were enrolled into the study.

### 2.2. Covariates

The main covariates of this retrospective study were as follows: (1) AC patient basic information: records of gender, age, and preoperative serum lipids levels; (2) AC patient pathologic information: records of tumor diameter, tumor differentiation, vascular invasion, pT, pN, and stage (pTNM); (3) AC patient treatment information: records including lymph nodes resection, postoperative complications (collected during 30 days after operation), and methods of postoperative adjuvant therapy. Postoperative data and survival status were obtained through multiple ways, including telephone reviews, outpatient follow-up, and the death registry system. Then, data of overall survival (OS, Time from operation to death) and recurrence-free survival (RFS, Time from operation to first-time recurrence) was acquired.

### 2.3. Statistical Analysis

For better visualization of statistical results, we conducted the statistical analyses in this study integrating SPSS (version 25.0 for windows) [18] and R (version 4.2.0 for windows) [19]. Categorical variables were summarized with frequency and percentage, and the Chi-square test or Fisher exact test were applied for the comparison of different groups. The optimal cutoff values of the serum lipids including TC, TG, HDL-C, and LDL-C were determined by ROC analysis. Univariate and multivariate Cox proportional hazard regression analyses were performed to identify the independent prognostic factors. All factors with *p* < 0.05 in univariate analyses were enrolled into the multivariate model. The Kaplan–Meier analyses were applied using the log-rank test, and the survival curves were depicted by R package “survminer”, “survival”, and “dplyr”. Weltch’t test was used for the comparison of serum lipids levels in different groups divided by various invasive factors (*, **, and *** represented *p* < 0.05, *p* < 0.01, and *p* < 0.001, respectively; *ns* represented that there was no significance). We evaluated statistical significance using two-sided tests, and *p* < 0.05 was considered statistically significant.

## 3. Results

### 3.1. Determination of Cutoff Values for Serum Lipids

The optimal cut-off values are found by weighting sensitivity and specificity. We performed ROC analysis and determined that the optimal cutoff values with joint maximum of sensitivity and specificity were 4.28 mmol/L for TC (AUC: 0.528, 95% CI: 0.453–0.602), 1.11 mmol/L for TG (AUC: 0.481, 95% CI: 0.407–0.556), 0.39 mmol/L for HDL-C (AUC: 0.561, 95% CI: 0.487–0.635), and 2.56 mmol/L for LDL-C (AUC: 0.589, 95% CI: 0.515–0.663), respectively. As such, we thereby divided the patients into high-lipid and low-lipid groups by the optimal cutoff values aforementioned. Additionally, the following analyses were conducted based on these groups.

### 3.2. Basic Characteristics of Patients

After screening according to inclusion and exclusion criteria, a total of 232 eligible AC patients were included in the study. All the data were collected until December 2020, and the median follow-up time was 34 months, with a range from 1 to 240 months. The baseline characteristics of these patients are summarized in Table 1. Their median age at diagnosis was 58 years (interquartile range, 49.75 to 65 years), and the ratio of male to female was 1.44. No less than 17 lymph nodes were resected in 69 (29.7%) patients. Concerning the pathology, a majority of patients had a moderately differentiated tumor (44.4%), and the mean tumor diameter of all patients was 2.40 cm. Among them, 101 (43.5%), 101 (43.5%), and 30 (12.9%) patients were staged in I, II, and III, respectively. In addition, 48 (20.7%) patients were confirmed with vascular invasion. After surgery, complications occurred in 91 (39.2%) patients and adjuvant therapy was performed in 59 (25.4%) patients. Furthermore, the descriptive data of preoperative serum lipids levels (TC, TG, HDL-C, and LDL-C) are shown in the Table 1. The details of postoperative complications and adjuvant therapy methods for AC patients undergoing curative pancreatoduodenectomy are summarized in the Table 2 and Table 3, respectively. According to these details, biliary/pancreatic fistula (31.9%), gastric emptying disorder (22.0%), and hemorrhage (18.7%) accounted for the majority of the postoperative complications. Regarding the adjuvant therapy, the overwhelming majority of these patients received chemotherapy (81.4%) in an adjuvant way. Then, we evaluated the severity of postoperative complications using the Clavien–Dindo system (Table 4), which demonstrated that a majority of patients with postoperative complications were in Grade II (60.4%). 

### 3.3. Association between Serum Lipids and Invasive Factors

**Figure 1:**
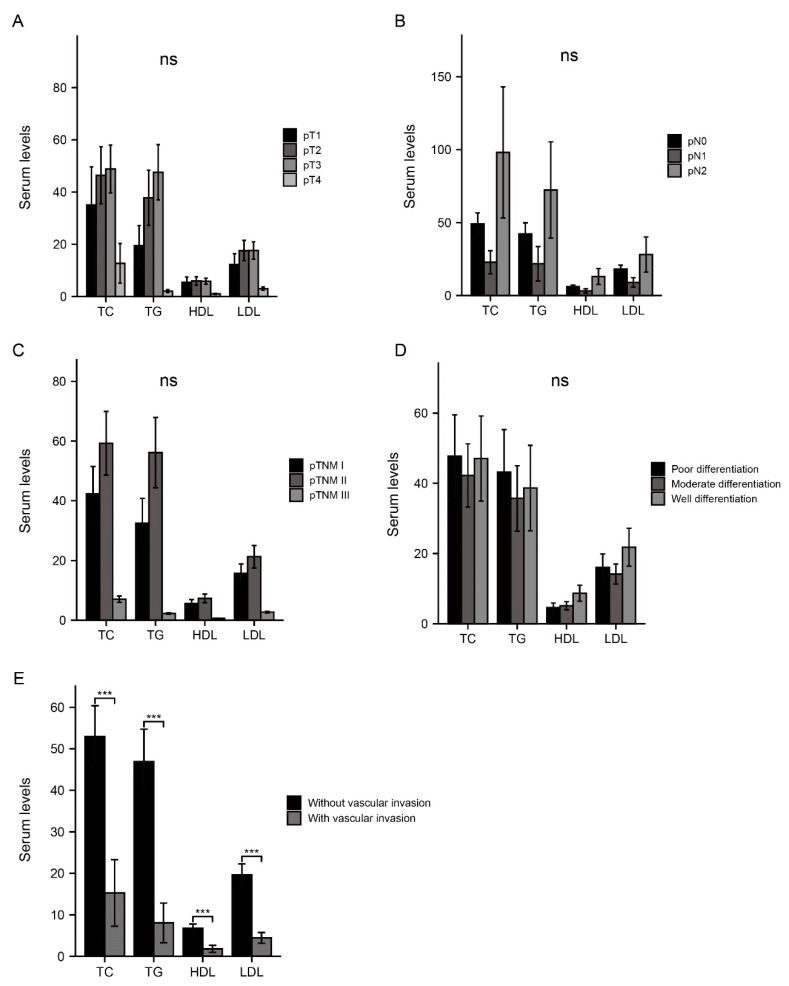
Correlation analysis between serum lipids and invasive factors. The serum levels of TC, TG, HDL, and LDL in different groups divided by pT (**A**), pN (**B**), pTNM (**C**), differentiation status (**D**), and vascular invasion status (**E**). ***, *p* < 0.001; ns, no significance.

In order to investigate the correlation of serum lipids with factors which make inroads on patient survival, patients in this retrospective study were further divided into different subgroups according to multiple invasive factors, including pT, pN, stage, tumor differentiation (grade), and vascular invasion. The serum levels of TC, TG, HDL-C, and LDL-C in each subgroup were compared to evaluate the association between serum lipids and invasive factors mentioned above. Among these factors, four types of serum lipids were observed to be significantly higher in patients without vascular invasion than those with (*p* < 0.001), implying a protective impact of serum lipids on reducing vascular invasion. Nevertheless, there was no statistical significance in the analysis for rest subgroups (Figure 1).

### 3.4. The Prognostic Role of Serum Lipids in OS and RFS

**Figure 2:**
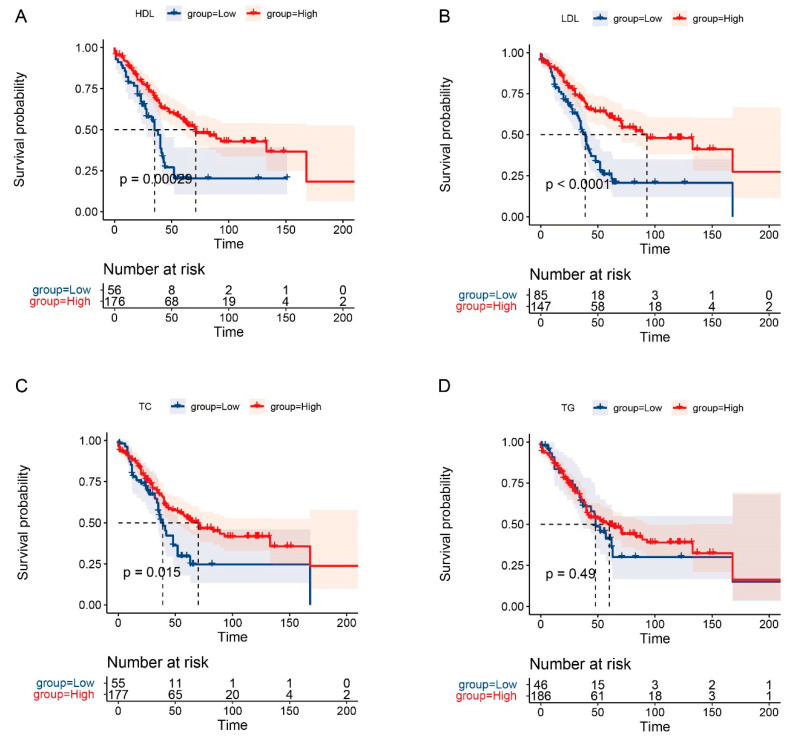
Survival analysis of OS for AC patients. KM curves of OS for HDL (**A**), LDL (**B**), TC (**C**), and TG (**D**).

**Figure 3:**
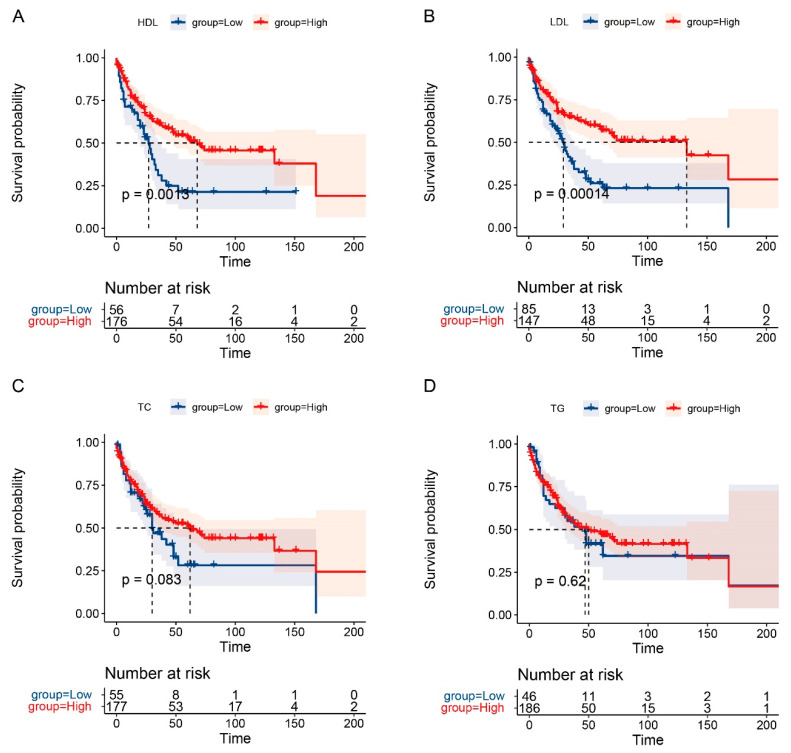
Survival analysis of RFS for AC patients. KM curves of RFS for HDL (**A**), LDL (**B**), TC (**C**), and TG (**D**).

The 1-year, 3-year, and 5-year OS rates for AC patients in this study were 86.1%, 64.1%, and 47.6%, respectively. Meanwhile, the 1-year, 3-year, and 5-year RFS rates were 75.8%, 54.8%, and 46.5%, respectively. Kaplan–Meier survival curves suggested that the levels of major serum lipids (TC, HDL-C, and LDL-C) were significantly associated with long-term prognosis of patients with AC (*p* < 0.05), except for TG. Furthermore, prolonged OS and RFS was obtained by AC patients with high levels of LDL-C (*p* < 0.01), the same was true for HDL-C as well (*p* < 0.01). In addition, patients with high levels of TC tended to own a better OS (*p* = 0.015) (Figure 2 and Figure 3). Moreover, the univariate and multivariate analysis identified that higher LDL-C level was a significant predictor for prolonged RFS of AC patients (HR = 0.43, 95% CI: 0.21–0.85, *p* = 0.015) (Table 5). Clavien–Dindo Grade of complications and T stage were also revealed to be independent prognostic factors for the long-term prognosis of AC patients (all *p* < 0.05) (Table 5 and Table 6).

## 4. Discussion

Lipid metabolism indicated by serum lipids (TC, TG, HDL-C, and LDL-C) is found to be involved in various important biological processes of cancer cells [20], and the association between serum lipids and cancers has sparked debate in recent years [21,22,23,24]. To the best of our knowledge, there is no study reported to have investigated the effects of serum lipids on the long-term prognosis of AC. In the present study, a high level of preoperative LDL-C was identified as a significant predictor for better RFS, which might produce a favorable impact on AC patients underwent curative surgical resection.

Previous studies and meta-analyses had investigated the roles of serum lipids (including TC, TG, HDL-C, and LDL-C) in the risk and prognosis of several cancer types. A significantly inverse association between high levels of serum lipids and risk of ovarian cancer [25], colorectal cancer [23], breast cancer [24], lung cancer [26], and non-hodgkin lymphoma [27] was identified. Additionally, a systematic review and meta-analysis demonstrated that serum TC (HR = 0.82, 95% CI: 0.75–0.90) and HDL-C (HR = 0.63, 95% CI: 0.47–0.86) were protective factors for long-term prognosis in patients with cancer [28]. As for LDL-C, previous studies revealed that patients with increased LDL-C seem to own a poor prognosis in lung cancer [29,30] and ovarian cancer [17,31], while a better prognosis was induced in gallbladder cancer [32]. In the current study, multiple analyses mainly demonstrated that high levels of LDL-C and HDL-C were significantly associated with a better survival of AC patients (*p* < 0.05).

Compared with previous studies, evidence seems to support the favorable impact of high HDL-C and LDL-C levels on AC patients in this study. From the perspective of mechanism research, the role of HDL-C in cancer prognosis was potentially related to the mechanisms as following. Firstly, tumor-associated macrophages (TAMs) include M1-TAM and M2-TAM that inhibits and promotes the tumor growth, respectively. HDL-C has an immunomodulatory effect of converting M1-TAM to M2-TAM [33]. In addition, HDL-C also induces multiple alterations at the cellular level, such as cholesterol exhaustion in TAM, thereby weakening the tumor promoting effect of TAM [34]. Moreover, serum lipids are observed to enhance the anti-inflammatory effect of neutrophil, the activation of CD8+T cell, and the survival of CD4+T cell, and inhibit tumor progression by strengthening the anti-tumor immune response [35]. Secondly, in inflammatory TME, HDL-C can suppress tubulogenesis and endothelial cell migration by downregulating the expression of VEGF, VEGFR2, and TNF-α, thereby inhibiting pathological angiogenesis [36]. In hypoxic TME, HDL-C can also promote the phosphorylation of VEGFR2 to enhance angiogenesis through the p38 MAPK signaling pathway [37]. Thirdly, in addition to anti-inflammatory and immune regulation, HDL-C also has antioxidant effects, which can maintain intracellular cholesterol homeostasis, regulate signal transduction and cell proliferation, thereby being a protective factor for cancer patients [38].

Regarding LDL-C, several studies have concluded that it had an unfavorable impact on the prognosis for cancer patients because of its oxidant effects [30], while views also exist that a low-level of LDL-C also tended to be associated with an increased cancer deaths [39]. The cause for perspectives of the latter originated from the observation in early retrospective epidemiological studies, such as the one from the Seven Countries Study published by Pekkanen et al., which demonstrated a reverse association between LDL-C and incidence of cancer [40]. Nevertheless, the early study from western society concerning this view had its limitations: the number of patients with low cholesterol levels was too limited to make the study balanced and comparable. Afterwards, a study based on the Chinese population was conducted by Chen et al. to investigate the same subject. They excluded patients dying in the first 3 years and the result demonstrated that patients with a lower cholesterol level had an increased incidence and mortality for liver cancer. In 2007, a larger scale study published by Iribarren et al. further completed a similar investigation. The cholesterol of 5941 men without history of coronary heart disease, stroke, cancer, and gastrointestinal-liver disease was prospectively tracked over 6 years. The risk factor adjusted all-cause mortality rate of those with lower cholesterol was demonstrated to increase by 30%. Meanwhile, the increased risk of mortality was analyzed to be induced by hematopoietic, esophageal, and prostate cancers and multiple non-cancer non-cardiovascular causes, particularly liver disease [41]. Multiple trials also drew a conclusion that there was a significantly inverse relationship between incidence of cancer and the achieved LDL-C level of patients taking statins [42]. This evidence functions as support for results of the present study. Additionally, LDL-C level was observed to be higher in AC patients without vascular invasion, suggesting that higher LDL-C level may reduce the risk of vascular invasion in our analysis, thereby accounting for the results as well.

There are also some limitations in this study that need to be taken into account. Firstly, this was a retrospective cohort study and therefore subject to various biases. Meanwhile, the sample size of this study was relatively small due to the rarity of AC. Secondly, except for major serum lipids including TC, TG, HDL-C, and LDL-C, several other alterations reflecting lipid metabolism were not included in the current study, such as Apo A1, Apo B, and Lp (a). The lack of these data may lead to a neglect of an opportunity to discover new insights. Thirdly, serum lipid levels were only tested preoperatively, so we were unable to further investigate the prognostic impact of changes in serum lipids dynamics. However, the strengths also should not be ignored that the present study was the first to discuss the impact of serum lipids on the prognosis of AC. In addition to the effects of serum lipids on the survival, we also compared the levels of serum lipids in different groups divided by various aggressive factors, and these differences may further suggest the reason for the impact of serum lipids on the prognosis of patients with AC. In addition, we controlled for the confounding factor of the application of lipid-lowering drugs, which makes our results more reliable. We hope this research can bring new insights in arrangement of AC patients to clinical workers.

## 5. Conclusions

A high level of preoperative LDL-C seems to produce a favorable impact on the long-term prognosis of ampullary adenocarcinoma (AC), which predicts a prolonged RFS for AC patients. Nevertheless, we demonstrated that the level of LDL-C was significantly higher in AC patients without vascular invasion, suggesting the possible impact of LDL-C on reducing the risk of vascular invasion.

## Figures and Tables

**Table 1:** Baseline characteristics of 232 AC patients.

Characteristics	Overall (*N* = 232)
Gender, *n* (%)	
Female	95 (40.9%)
Male	137 (59.1%)
Age, year	
Media (interquartile range)	58 (49.75–65)
Tumor diameter, cm	
Mean (SD)	2.40 (1.10)
Tumor differentiation, *n* (%)	
Poor differentiation	78 (33.6%)
Moderate differentiation	103 (44.4%)
Well differentiation	51 (22.0%)
Lymph nodes resection, *n* (%)	
<17	163 (70.3%)
≥17	69 (29.7%)
Vascular invasion, *n* (%)	
No	184 (79.3%)
Yes	48 (20.7%)
pT, *n* (%)	
T1	38 (16.4%)
T2	79 (34.1%)
T3	111 (47.8%)
T4	4 (1.7%)
pN, *n* (%)	
N0	169 (72.8%)
N1	53 (22.8%)
N2	10 (4.3%)
Stage, *n* (%)	
I	101 (43.5%)
II	101 (43.5%)
III	30 (12.9%)
Postoperative complication, *n* (%)	
No	141 (60.8%)
Yes	91 (39.2%)
Postoperative adjuvant therapy, *n* (%)	
No	173 (74.6%)
Yes	59 (25.4%)
TC, *n* (%)	
<4.28 mmol/L	55 (23.7%)
≥4.28 mmol/L	177 (76.3%)
TG, *n* (%)	
<1.11 mmol/L	46 (19.8%)
≥1.11 mmol/L	186 (80.2%)
HDL-C, *n* (%)	
<0.39 mmol/L	56 (24.1%)
≥0.39 mmol/L	176 (75.9%)
LDL-C, *n* (%)	
<2.56 mmol/L	85 (36.6%)
≥2.56 mmol/L	147 (63.4%)

**Table 2:** The details of postoperative complications for 91 AC patients undergoing curative pancreatoduodenectomy.

Complication	Overall (*N* = 91)
Abdominal infection	11 (12.1%)
Abdominal infection and pancreatic fistula	2 (2.2%)
Anastomotic leakage	1 (1.1%)
Biliary/pancreatic fistula	29 (31.9%)
Gastric emptying disorder	20 (22.0%)
Hemorrhage	17 (18.7%)
Hemorrhage and abdominal infection	1 (1.1%)
Hemorrhage and pancreatic fistula	1 (1.1%)
Hemorrhage, gastric emptying disorder, and abdominal infection	1 (1.1%)
Intestinal fistula and abdominal infection	1 (1.1%)
Lung infection	5 (5.5%)
pancreatic fistula and incision fat liquefaction	1 (1.1%)
Urinary system infection	1 (1.1%)

**Table 3:** The details of postoperative adjuvant therapy methods for 59 AC patients undergoing curative pancreatoduodenectomy.

Adjuvant Therapy Method	Overall (*N* = 59)
Chemotherapy	48 (81.4%)
Combination of chemotherapy and radiation	2 (3.4%)
Combination of chemotherapy and targeted therapy	1 (1.7%)
Continuous hyperthermic peritoneal perfusion	1 (1.7%)
Radiation	4 (6.8%)
Unknown	3 (5.1%)

**Table 4:** Clavien–Dindo system analysis of ampullary adenocarcinoma.

Clavien–Dindo System for Comlication	Overall (*N* = 91)
Grade I	1 (1.1%)
Grade II	55 (60.4%)
Grade III	29 (31.9%)
Grade IV	6 (6.6%)

**Table 5:** Univariate and multivariate analysis of ampullary adenocarcinoma with RFS.

Variate	Univariate Analysis			Multivariate Analysis		
	HR	95%CI	*p*	HR	95%CI	*p*
Gender (male vs. female)	1.08	0.74–1.58	0.683			
Age	1.02	1–1.04	0.067			
Year of inclusion (after 2010 vs. before 2010)	1.16	0.79–1.7	0.440			
Tumor diameter	1.15	0.96–1.38	0.118			
Differentiation (well vs. non-well)	0.61	0.38–0.99	0.044	1.02	0.46–2.24	0.965
Lymph nodes resection (≥17 vs. <17)	1.07	0.72–1.61	0.73			
Vascular invasion (yes vs. no)	1.64	1.05–2.54	0.029	0.77	0.32–1.83	0.555
pT (T3, T4 vs. T1, T2)	2.19	1.5–3.19	<0.001	1.71	0.88–3.34	0.116
pN (N1, N2 vs. N0)	2.28	1.54–3.39	<0.001	1.6	0.55–2.5	0.682
Postoperative complication (yes vs. no)	1.31	0.9–1.92	0.157			
Clavien–Dindo system (grade III/IV vs. I/II)	2.18	1.21–3.92	0.009	2.16	1.16–4.02	0.015
Postoperative adjuvant therapy (yes vs. no)	1.21	0.79–1.84	0.384			
TC (≥4.28 vs. <4.28)	0.69	0.46–1.05	0.082			
TG (≥1.11 vs. <1.11)	0.9	0.58–1.39	0.624			
HDL-C (≥0.39 vs. <0.39)	0.52	0.35–0.78	0.002	1.2	0.59–2.45	0.620
LDL-C (≥2.56 vs. <2.56)	0.49	0.33–0.71	<0.001	0.43	0.21–0.85	0.015

**Table 6:** Univariate and multivariate analysis of ampullary adenocarcinoma with OS.

Variate	Univariate Analysis			Multivariate Analysis		
	HR	95%CI	*p*	HR	95%CI	*p*
Gender (male vs. female)	1.14	0.78–1.66	0.507			
Age	1.02	1–1.04	0.031	1.03	1–1.06	0.084
Year of inclusion (after 2010 vs. before 2010)	1.22	0.84–1.79	0.296			
Tumor diameter	1.1	0.92–1.31	0.288			
Differentiation (well vs. non-well)	0.64	0.39–1.03	0.064			
Lymph nodes resection (≥17 vs. <17)	1.15	0.77–1.72	0.508			
Vascular invasion (yes vs. no)	1.53	0.99–2.38	0.057			
pT (T3, T4 vs. T1, T2)	2.15	1.47–3.14	<0.001	2.04	1.04–4.01	0.037
pN (N1, N2 vs. N0)	2.2	1.48–3.26	<0.001	0.87	0.43–1.77	0.704
Postoperative complication (yes vs. no)	1.32	0.9–1.93	0.15			
Clavien–Dindo system (III/IV vs. I/II)	2.13	1.19–3.81	0.011	2.14	1.16–3.95	0.015
Postoperative adjuvant therapy (yes vs. no)	1.16	0.76–1.76	0.502			
TC (≥4.28 vs. <4.28)	0.6	0.39–0.91	0.016	0.54	0.25–1.17	0.116
TG (≥1.11 vs. <1.11)	0.86	0.55–1.33	0.495			
HDL-C (≥0.39 vs. <0.39)	0.48	0.32–0.72	<0.001	1.03	0.5–2.15	0.930
LDL-C (≥2.56 vs. <2.56)	0.44	0.3–0.65	<0.001	0.54	0.24–1.2	0.129

## Data Availability

All data analyzed in this study can be available from the corresponding author.

## References

[1] Okano K., Oshima M., Yachida S., Kushida Y., Kato K., Kamada H., Wato M., Nishihira T., Fukuda Y., Maeba T. (2014). Factors predicting survival and pathological subtype in patients with ampullary adenocarcinoma. J. Surg. Oncol..

[2] Rizzo A., Dadduzio V., Lombardi L., Ricci A.D., Gadaleta-Caldarola G. (2021). Ampullary Carcinoma: An Overview of a Rare Entity and Discussion of Current and Future Therapeutic Challenges. Curr. Oncol..

[3] Ahn D.H., Bekaii-Saab T. (2014). Ampullary cancer: An overview. Am. Soc. Clin. Oncol. Educ. Book.

[4] Albores-Saavedra J., Schwartz A.M., Batich K., Henson D.E. (2009). Cancers of the ampulla of vater: Demographics, morphology, and survival based on 5625 cases from the SEER program. J. Surg. Oncol..

[5] Cheng C., Geng F., Cheng X., Guo D. (2018). Lipid metabolism reprogramming and its potential targets in cancer. Cancer Commun. (Lond.).

[6] Snaebjornsson M.T., Janaki-Raman S., Schulze A. (2020). Greasing the Wheels of the Cancer Machine: The Role of Lipid Metabolism in Cancer. Cell Metab..

[7] Bian X., Liu R., Meng Y., Xing D., Xu D., Lu Z. (2021). Lipid metabolism and cancer. J. Exp. Med..

[8] Luo X., Cheng C., Tan Z., Li N., Tang M., Yang L., Cao Y. (2017). Emerging roles of lipid metabolism in cancer metastasis. Mol. Cancer.

[9] Cao Y. (2019). Adipocyte and lipid metabolism in cancer drug resistance. J. Clin. Investig..

[10] Merino Salvador M., Gómez de Cedrón M., Moreno Rubio J., Falagán Martínez S., Sánchez Martínez R., Casado E., Ramírez de Molina A., Sereno M. (2017). Lipid metabolism and lung cancer. Crit. Rev. Oncol. Hematol..

[11] Alannan M., Fayyad-Kazan H., Trézéguet V., Merched A. (2020). Targeting Lipid Metabolism in Liver Cancer. Biochemistry.

[12] Cedó L., Reddy S.T., Mato E., Blanco-Vaca F., Escolà-Gil J.C. (2019). HDL and LDL: Potential New Players in Breast Cancer Development. J. Clin. Med..

[13] Stoykova G.E., Schlaepfer I.R. (2019). Lipid Metabolism and Endocrine Resistance in Prostate Cancer, and New Opportunities for Therapy. Int. J. Mol. Sci..

[14] Nam S.Y., Park B.J., Nam J.H., Kook M.C. (2019). Effect of Helicobacter pylori eradication and high-density lipoprotein on the risk of de novo gastric cancer development. Gastrointest. Endosc..

[15] Pakiet A., Kobiela J., Stepnowski P., Sledzinski T., Mika A. (2019). Changes in lipids composition and metabolism in colorectal cancer: A review. Lipids Health Dis..

[16] Gong J., Lin Y., Zhang H., Liu C., Cheng Z., Yang X., Zhang J., Xiao Y., Sang N., Qian X. (2020). Reprogramming of lipid metabolism in cancer-associated fibroblasts potentiates migration of colorectal cancer cells. Cell Death Dis..

[17] Lin Q., Liu W., Xu S., Sun L. (2022). Associations of preoperative serum high-density lipoprotein cholesterol and low-density lipoprotein cholesterol levels with the prognosis of ovarian cancer. Arch. Gynecol. Obstet..

[18] SPSS: IBM Corp (2017). IBM SPSS Statistics for Windows, Version 25.5.

[19] R Core Team (2022). R: A Language and Environment for Statistical Computing.

[20] Santos C.R., Schulze A. (2012). Lipid metabolism in cancer. FEBS J..

[21] Radišauskas R., Kuzmickienė I., Milinavičienė E., Everatt R. (2016). Hypertension, serum lipids and cancer risk: A review of epidemiological evidence. Medicina (Kaunas Lithuania).

[22] Kitahara C.M., Berrington de González A., Freedman N.D., Huxley R., Mok Y., Jee S.H., Samet J.M. (2011). Total cholesterol and cancer risk in a large prospective study in Korea. J. Clin. Oncol..

[23] Van Duijnhoven F.J., Bueno-De-Mesquita H.B., Calligaro M., Jenab M., Pischon T., Jansen E.H., Frohlich J., Ayyobi A., Overvad K., Toft-Petersen A.P. (2011). Blood lipid and lipoprotein concentrations and colorectal cancer risk in the European Prospective Investigation into Cancer and Nutrition. Gut.

[24] Törnberg S.A., Holm L.E., Carstensen J.M. (1988). Breast cancer risk in relation to serum cholesterol, serum beta-lipoprotein, height, weight, and blood pressure. Acta Oncol. (Stockholm Sweden).

[25] Onwuka J.U., Okekunle A.P., Olutola O.M., Akpa O.M., Feng R. (2020). Lipid profile and risk of ovarian tumours: A meta-analysis. BMC Cancer.

[26] Lyu Z., Li N., Wang G., Feng X., Chen S., Su K., Li F., Wei L., Li X., Xie S. (2019). Independent and joint associations of blood lipids and lipoproteins with lung cancer risk in Chinese males: A prospective cohort study. Int. J. Cancer.

[27] Lim U., Gayles T., Katki H.A., Stolzenberg-Solomon R., Weinstein S.J., Pietinen P., Taylor P.R., Virtamo J., Albanes D. (2007). Serum high-density lipoprotein cholesterol and risk of non-hodgkin lymphoma. Cancer Res..

[28] Zhou P., Li B., Liu B., Chen T., Xiao J. (2018). Prognostic role of serum total cholesterol and high-density lipoprotein cholesterol in cancer survivors: A systematic review and meta-analysis. Clin. Chim. Acta.

[29] Liu T., Zhou T., Luo F., Yang Y., Zhao S., Huang Y., Zhao H., Zhang L., Zhao Y. (2021). Clinical Significance of Kinetics of Low-Density Lipoprotein Cholesterol and Its Prognostic Value in Limited Stage Small Cell Lung Cancer Patients. Cancer Control.

[30] Zhou T., Zhan J., Fang W., Zhao Y., Yang Y., Hou X., Zhang Z., He X., Zhang Y., Huang Y. (2017). Serum low-density lipoprotein and low-density lipoprotein expression level at diagnosis are favorable prognostic factors in patients with small-cell lung cancer (SCLC). BMC Cancer.

[31] Li A.J., Elmore R.G., Chen I.Y., Karlan B.Y. (2010). Serum low-density lipoprotein levels correlate with survival in advanced stage epithelial ovarian cancers. Gynecol. Oncol..

[32] Yuan B., Fu J., Yu W.L., Fu X.H., Qiu Y.H., Yin L., Zhu B., Zhang Y.J. (2019). Prognostic value of serum high-density lipoprotein cholesterol in patients with gallbladder cancer. Rev. Esp. De Enferm. Dig. Organo Off. De La Soc. Esp. De Patol. Dig..

[33] Zamanian-Daryoush M., Lindner D., Tallant T.C., Wang Z., Buffa J., Klipfell E., Parker Y., Hatala D., Parsons-Wingerter P., Rayman P. (2013). The cardioprotective protein apolipoprotein A1 promotes potent anti-tumorigenic effects. J. Biol. Chem..

[34] Smythies L.E., White C.R., Maheshwari A., Palgunachari M.N., Anantharamaiah G.M., Chaddha M., Kurundkar A.R., Datta G. (2010). Apolipoprotein A-I mimetic 4F alters the function of human monocyte-derived macrophages. Am. J. Physiol. Cell Physiol..

[35] Zhao T.J., Zhu N., Shi Y.N., Wang Y.X., Zhang C.J., Deng C.F., Liao D.F., Qin L. (2021). Targeting HDL in tumor microenvironment: New hope for cancer therapy. J. Cell. Physiol..

[36] Bursill C.A., Castro M.L., Beattie D.T., Nakhla S., van der Vorst E., Heather A.K., Barter P.J., Rye K.A. (2010). High-density lipoproteins suppress chemokines and chemokine receptors in vitro and in vivo. Arterioscler. Thromb. Vasc. Biol..

[37] Pagès G., Berra E., Milanini J., Levy A.P., Pouysségur J. (2000). Stress-activated protein kinases (JNK and p38/HOG) are essential for vascular endothelial growth factor mRNA stability. J. Biol. Chem..

[38] Mazzuferi G., Bacchetti T., Islam M.O., Ferretti G. (2021). High density lipoproteins and oxidative stress in breast cancer. Lipids Health Dis..

[39] Pekkanen J., Nissinen A., Vartiainen E., Salonen J.T., Punsar S., Karvonen M.J. (1994). Changes in serum cholesterol level and mortality: A 30-year follow-up. The Finnish cohorts of the seven countries study. Am. J. Epidemiol..

[40] Jacobs D., Blackburn H., Higgins M., Reed D., Iso H., McMillan G., Neaton J., Nelson J., Potter J., Rifkind B. (1992). Report of the Conference on Low Blood Cholesterol: Mortality Associations. Circulation.

[41] Stein E.A., Raal F.J. (2014). Targeting LDL: Is lower better and is it safe. Best Pract. Res. Clin. Endocrinol. Metab..

[42] Alsheikh-Ali A.A., Maddukuri P.V., Han H., Karas R.H. (2007). Effect of the magnitude of lipid lowering on risk of elevated liver enzymes, rhabdomyolysis, and cancer: Insights from large randomized statin trials. J. Am. Coll. Cardiol..

